# Supplementary material for: Are carbon emissions trading and green financial instruments synergistic? -Comprehensive quantitative research based on content analysis
Source: PLoS One. 2024 Mar 7;19(3):e0298601. doi: 10.1371/journal.pone.0298601 (PMC10919601; doi:10.1371/journal.pone.0298601)
Supplement: S1 Data — (ZIP) [file pone.0298601.s001.zip › Data/地级市Z_F工作报告.zip!/╕╩╦α/┬ñ─╧╩╨/└┤╫╘╣1⁄2╓┌║┼í╛┬φ┐╦╩2╛▌═°í┐_2012.docx]

各位代表：

现在，我代表市人民政府向大会作工作报告，请予审议，并请各位政协委员和列席人员提出意见。

本届政府工作回顾

市二届一次人代会以来,我们走过了极为特殊、极其艰难、极不平凡的五年。在即将过去的五年里,我们在省委、省政府和市委的坚强领导下，在市人大、市政协的监督支持下，坚持以科学发展观为指导，认真贯彻落实市委总体思路和工作部署，团结带领全市各族人民，积极应对各种困难、挑战和重大自然灾害，坚定信心、抢抓机遇、攻坚克难、苦干实干，圆满完成了本届政府确定的各项目标任务，为今后发展打下了坚实基础。

——经济发展速度加快，综合实力明显增强。我们克服国际金融危机的严重影响，抢抓灾后重建和国家实施西部大开发、支持甘肃经济社会发展、扩大内需等一系列重大机遇，全力加快经济发展，实现了经济总量和速度、效益的同步增长。到今年12月底,预计全市生产总值由2006年的93亿元增加到190亿元，年均增长10.7%，人均达到7010元，年均增长11.1%。全社会固定资产投资由2006年的36亿元增加到295亿元，年均增长52%。大口径财政收入由2006年的11.6亿元增加到27亿元，年均增长18.5%；市县级财政收入由2006年的3.9亿元增加到12.2亿元，年均增长25.7%。社会消费品零售总额由2006年的21.9亿元增加到49亿元，年均增长17.5%。城镇居民人均可支配收入由2006年的7071元增加到12056元，年均增长11.3%；农民人均纯收入由2006年的1464元增加到2712元，年均增长13.1%。

——灾后重建取得全面胜利，城乡面貌发生历史性巨变。2008年发生了百年不遇的“5.12”特大地震，陇南是甘肃受灾最严重的地区，在党中央、国务院的亲切关怀和省委、省政府的坚强领导下，在部队官兵、民兵预备役人员及社会各界特别是深圳市的无私援助下，我们快速反应、沉着应对，迅速组织开展抢险救灾，最大限度地减少了人员伤亡和财产损失，取得了抗震救灾的全面胜利。灾后重建开始后，我们按照省委、省政府和市委的总体部署，制定出台了推进灾后重建有序开展的30个指导性文件，在不同阶段深入研究重大问题，不断完善符合陇南实际的重建思路，分阶段有序推进灾后重建，2011年9月底全面完成了城乡居民住房维修重建和学校、医院等民生工程。全市累计实施灾后重建项目3905个，完成投资391亿元，占已落实资金项目总投资的105.1%。全市73%的农户搬入了安全舒适的新居。实施教育、卫生、文化、体育等公共服务设施重建项目1628个，建成学校549所、医院257所，使长期落后的公共服务设施得到有效改善，服务功能得到极大提升。集中实施了城乡基础设施、产业重组、市场体系、防灾减灾等项目，为全市经济社会跨越式发展奠定了坚实基础。同时，扎实有效开展了“8.12”暴洪灾害应急抢险和灾后恢复重建，取得了阶段性成效。

——项目建设成效显著，投资规模高幅增长。我们把项目建设作为加快发展的引擎，积极争取和认真实施各类项目，全市项目建设实现了重大突破。累计争取国家正常投资60亿元，立项建设各类项目3616个，完成投资875亿元，是上一个五年投资总和130亿元的6.7倍。特别是在陇南境内全长239公里投资210亿元的兰渝铁路、全长130公里投资117亿元的武都至罐子沟高速公路和全长91公里投资121亿元的成县至武都高速公路等重大项目相继开工建设，陇南落后的交通面貌将发生历史性巨变。十堰至天水高速公路陇南段、陇南成州民用机场、渭源至武都高速公路及一批基础设施项目前期工作进展顺利。集中实施了市列重点项目738个，累计完成投资317亿元。成州公司10万吨锌冶炼、麒麟寺水电站、金徽酒业集团公司1.5万吨白酒生产线扩建、武都和成县两个百万吨干法水泥生产线等一批重大工业项目建成投产，苗家坝、橙子沟水电站等重大项目建设顺利推进。累计签约招商引资项目758个，到位资金194亿元，合同履约率达到85%，招商引资工作取得新成效。

——经济结构不断优化，发展能力得到增强。我们分别召开并认真贯彻全市工业、特色产业和旅游发展大会精神，围绕增强综合经济实力，着力调整经济结构、转变发展方式，三次产业得到快速发展。一是特色农业产业化步伐加快。特色农业规模扩大、效益提高，核桃、花椒、油橄榄、中药材、苹果等种植面积达到911万亩，比2006年增加263万亩；肉类产量达到12万吨，比2006年增加3.6万吨；粮食生产连续五年丰收，产量达到102万吨，比2006年增加26万吨。农业龙头企业、农民专合组织、农业专业市场快速发展，带动农村经济发展的能力进一步增强。积极开展开发式扶贫，累计投入扶贫资金21亿元，减少贫困人口28万人，农村贫困面下降了13.7个百分点。累计输转劳务327万人，创劳务收入218亿元。二是工业经济发展壮大。工业经济在金融危机和地震灾害的双重压力下实现快速恢复性增长，规模以上工业增加值达到28亿元；独一味、西部资源两家企业成功上市，成州公司、宝徽公司、独一味公司跻身甘肃工业100强，完成了厂坝矿区资源整合、资产重组工作，整合后新组建的甘肃厂坝有色金属有限责任公司已投入运行，铅锌产业开发迈上了新的台阶；徽县工业集中区、西成开发区和其它县区工业集中区建设积极推进。三是第三产业快速发展。交通运输、商贸流通、旅游、房地产开发、金融保险、社会服务等产业持续活跃，第三产业增加值达到84亿元，年均增长12.4%，第三产业对经济的贡献率明显提升。

——基础设施建设加快，发展条件明显改善。我们着力破解制约经济社会发展的瓶颈，千方百计改善基础设施、提升发展条件，进一步夯实了城乡发展基础。一是城乡建设进程加快。我们适时召开并认真贯彻全市城市建设和新农村建设现场会议精神，推广礼县、两当经验，加快推进城市建设和新农村建设，取得了突破性进展，全市城镇化率达到24%，比2006年提高了6.5个百分点。市区东江新区、钟楼滩新区开发建设和旧城区改造取得重大进展，长江大道东西延伸工程全面开工建设，城市规模进一步扩大；八县县城和重点建制镇集中实施了一批市政设施和公共服务设施项目，建成生态文明新农村553个，呈现出各具特色、协调发展的良好势头。二是交通落后状况显著改善。大力实施了国省干线公路升等改造、农村公路通达通畅工程，累计改造和新建等级公路1931条11566公里，建成便民桥400座。全市公路里程由8933公里增加到14465公里，等级公路里程由3339公里增加到12878公里，通油路乡镇由84个增加到169个，新增通公路行政村1583个。三是水利建设力度加大。实施重大水利项目1154项，新增有效灌溉面积19万亩，发展补灌面积20万亩，新修梯田53万亩，治理水土流失1703平方公里；大力实施人饮安全工程，累计解决了50万人的饮水不安全问题。四是生态环境明显优化。完成退耕还林41万亩,实施天然林保护工程1014万亩、生态公益林321万亩、绿色通道建设2200公里，全市森林覆盖率由38.9%提高到42.4%。节能减排任务全面完成，重点区域、重点企业污染防治取得实效，农村生态保护与环境综合整治稳步推进。五是能源通信建设得到加强。农网改造全面完成，建成330千伏变电站4座、110千伏变电站8座，新增发电装机容量24万千瓦；通讯邮政行业迅速发展，实现了服务网络全覆盖，服务质量和效果明显提高。

——民生建设取得实效，社会事业全面发展。我们坚持以人为本，在加快经济发展的同时，高度关注民生，加快发展社会事业，使人民生活水平得到明显提高。九县区全部实现了“两基”目标，陇南师专顺利通过教育部人才培养水平评估，高考二本以上录取人数累计达到12364人，比上一个五年增加6803人；医疗卫生服务体系逐步完善，新农合参合率达到96.7%，筹资水平提高到230元，国家基本药物制度在基层医疗机构全面实施，公共卫生服务水平和城乡居民健康水平有所提高。完成科技培训196万人次，取得科技成果99项。建成了一批广播电视“村村通”、县区“三馆”和乡镇综合文化站、文化大院、农家书屋；人口自然增长率控制在6.38‰以内；新增城镇就业8.6万人，城镇登记失业率控制在4%以内。城乡居民社会养老保险试点工作启动，最低生活保障和医疗保险、失业保险、工伤保险覆盖面逐年扩大。安全生产、食品药品监管、社会治安综合治理得到不断加强，群众信访合理问题基本得到解决，社会大局保持和谐稳定。坚持为群众办实事，逐年解决了一些影响群众生产生活的具体问题。文物和非物质文化遗产保护、民族宗教、外事侨务、统计、档案、地方志、气象、防灾减灾、“双拥”、人防、民兵预备役和妇女儿童、残疾人、老年人、慈善、红十字会事业取得了新进步。

——自身建设得到加强，行政效能不断提升。我们适应新形势、新任务的要求，坚持将政府自身建设摆在突出位置。围绕转变政府职能，深入推进政府机构改革、教育教学改革、财税体制改革、农村及涉农领域各项改革，初步形成了有利于科学发展的体制机制；围绕推进依法行政，认真落实行政执法责任制，完善行政执法监督制度、行政复议制度、政务公开制度、行政审批制度、规范性文件备案审查制度，对行政执法主体进行了全面清理，及时审理行政复议事项，加快了法制化规范化进程。围绕加强廉政建设，加快推进惩防体系建设，严格执行《廉政准则》，廉政建设责任制得到有效落实；严格落实灾后重建、扩大内需、政府投资项目资金监管措施，深入开展工程领域突出问题、“小金库”等各类专项治理活动，及时发现、纠正和查处了一些违规违纪行为；广泛开展政风行风评议活动，机关作风有了明显好转。

各位代表，2011年是本届政府任期的最后一年。我们认真贯彻落实省委、省政府和市委的各项决策部署，按照既定的目标任务，狠抓工作落实，全面或超额完成了市二届六次人代会确定的主要预期目标。预计全年生产总值比2010年增长12.3%；全社会固定资产投资增长20%；社会消费品零售总额增长19%；大口径财政收入增长31%，市县级财政收入增长30.7%；城镇居民人均可支配收入增长13.5%，农民人均纯收入增长18%。

各位代表，在即将过去的五年里，全市人民同心同德，同舟共济，迎难而上，真抓实干，在各种困难、挑战和自然灾害面前自强不息、勇创一流，经济社会发展取得了显著成就，陇南经济社会各个方面都发生了深刻变化，我们已经站在了新的历史起点上！回顾五年的发展历程，我们深切感受到成绩来之不易。陇南的每一个发展变化，都得益于省委、省政府和市委的坚强领导，得益于市人大、市政协和社会各界的监督支持，得益于全市人民的团结一心、共同奋斗，得益于全国人民尤其是深圳人民的无私援助。在此，我代表市人民政府，向全市广大干部群众，向中央、省驻陇南各单位、人民解放军和武警驻陇南部队官兵，向各民主党派、工商联、各人民团体、无党派人士、离退休老同志及所有关心、支持陇南发展的社会各界人士表示崇高的敬意和衷心的感谢！

各位代表,通过五年来的成功实践与艰苦探索，我们主要有以下五个方面的体会和启示：一是始终坚持党的领导是做好政府工作的关键所在。新一届市委领导班子准确把握陇南发展的总体特征和阶段性特征，适时调整完善工作思路，为做好政府工作提供了政治保证。我们始终坚持市委的坚强领导，认真贯彻落实市委各项决策部署，自觉维护市委权威，把市委的总体思路和决策部署在政府工作实践中不断深化拓展，切实增强了做好各项工作的执行力。二是始终坚持人大政协的监督支持是做好政府工作的重要前提。近年来，市人大、市政协充分履行职能，寓支持于监督之中，积极支持政府工作，人大代表、政协委员立足岗位实际，提出了许多珍贵的意见建议，为政府工作提供了强有力的帮助。我们积极主动向人大及其常委会报告工作，自觉接受人大依法监督和工作监督，主动接受政协民主监督，认真听取各种意见建议，使政府工作切实得到了改进和加强。三是始终坚持执政为民是做好政府工作的根本要求。我们牢记全心全意为人民服务的宗旨，把实现好、维护好、发展好最广大人民的根本利益，作为政府工作的出发点和落脚点，坚持每年为群众办实事、办好事，尽最大努力解决群众最关心、最直接、最现实的利益问题，得到了广大人民群众的拥护支持。四是始终坚持创新方式、狠抓落实是做好政府工作的重要途径。我们始终抢抓各种机遇，把积极争取国家项目投资和资金支持作为发展的重要抓手，创新项目生成机制，狠抓了各类重大项目的实施，形成各级各部门齐心协力抓项目的工作格局；把研究解决经济社会发展中的重大问题作为推动发展的重要手段，不断破解发展难题，提高了科学发展水平，尤其在灾后重建中，探索出了符合陇南实际的重建路子，并实行领导干部包抓重建项目责任制，确保了灾后重建任务的顺利完成。五是始终坚持依法行政是做好政府工作的基本保证。我们坚持推进依法行政，健全行政执法责任制和权力运行监督制约机制，用制度管权，靠制度管人，按制度办事，查处各类不正之风和腐败行为；全面推进政务公开，规范办事程序，提高了政府工作的透明度和群众信任度。所有这些，都需要我们在今后五年的政府工作中继续坚持。

在肯定成绩的同时，我们也清醒地认识到，五年来，虽然全市经济得到了长足发展、综合实力得到极大提升，但由于经济总量小、人均水平低，综合实力与省内先进地区相比仍然有较大差距；虽然农业特色产业基地已初具规模，但由于新品种、新技术利用率低，龙头企业数量少，营销体系不健全，品牌建设滞后，质量效益不高的问题比较突出，提质增效仍然是农业特色产业发展面临的首要问题；虽然工业经济取得了一定发展，但基础薄弱，以矿业开发为主体，产业结构单一，企业规模小，产品市场竞争力不强，缺少推动县域经济发展和促进产业优化升级的大项目、好项目，增加工业经济总量、提高发展水平仍然是今后工业发展的主要任务；虽然在灾后重建中实施了一批防灾减灾项目，但由于陇南地质结构复杂和降雨时空分布不均，暴洪、泥石流灾害易发多发仍然是威胁全市人民的心腹大患；虽然通过改革创新，政府工作效能有所提升，但是发展环境还不够宽松，有的部门工作作风不实、服务意识不强，个别人员存在不廉洁行为，影响了政府的形象，加强政府自身建设仍然十分迫切。对此，我们一定高度重视，采取切实有效的措施，认真加以解决。

今后五年的目标和主要任务

各位代表，未来五年，是我市深入实施“十二五”规划的关键时期。今后五年的发展，是在灾后重建奠定良好基础上的再发展，尽管存在各种困难和挑战，但也面临一系列重大发展机遇。市第三次党代会审时度势提出的未来五年全市经济社会发展的指导思想、奋斗目标和重大举措，为做好新一届政府工作提供了行动纲领和政治保证。站在新起点，谋求新发展，我们必须与时俱进、开拓创新，振奋精神、团结奋斗，在破解难题中加快发展，在化解矛盾中促进和谐，以一年一个新台阶，五年实现新跨越的雄心壮志，奋力推进陇南科学发展！

今后五年政府工作的指导思想是：以邓小平理论和“三个代表”重要思想为指导，深入实践科学发展观，认真贯彻落实市委总体发展思路，以富民强市为目标，围绕发展与和谐两大主题，抢抓国家深入实施西部大开发战略、支持甘肃经济社会发展、建设关中—天水经济区等重大历史机遇，实现交通建设、工业发展、城镇建设三大突破，突出抓好特色农业、旅游开发、生态建设、社会事业、改善民生五个重点，强化项目带动、资源整合、优化环境、创新机制和党对政府工作的领导五项保证，努力推动陇南经济社会实现跨越式发展。

按照市第三次党代会确定的奋斗目标，未来五年全市要力争实现“四个翻番、四个大幅增长”，即到2016年生产总值、固定资产投资、财政收入、城镇建成区面积比2011年翻一番；工业增加值、社会消费品零售总额、城镇居民人均可支配收入、农民人均纯收入实现大幅度增长。到2016年，生产总值总量达到380亿元,年均增长13%以上；工业增加值达到84亿元，年均增长18%以上；全社会固定资产投资达到647亿元，年均增长20%以上；财政收入达到54亿元,年均增长15%以上；社会消费品零售总额达到112亿元，年均增长18%以上；城镇居民人均可支配收入年均增长12%以上；农民人均纯收入年均增长13%以上；城镇建成区面积达到100平方公里。实现经济更加繁荣，产业更加壮大，基础更加坚实，生态更加良好，民生更加改善，社会更加和谐。

围绕实现上述目标，必须在以下六个方面实现新跨越：

（一）着力转变发展方式，实现经济实力大提升。坚持调整优化经济结构，努力加快工业、特色农业和第三产业发展，不断增强综合实力。一是加快特色农业提质增效。围绕建大基地、育大龙头、兴大产业、占大市场的思路，加快特色农业产业化进程，按照“四产区、五基地、多片带”的发展布局，以规模化发展、标准化建园为重点，着力培育一批基地面积达到千亩以上的专业村、万亩以上的示范乡镇，基本实现基地建设在最佳适宜区合理覆盖，力争到2016年全市特色产业面积达到并稳定在1000万亩左右。建立适应农业特色产业发展的科技推广体系，并组建技术研发中心，努力为特色农业提供科技服务。加强产业内部结构调整，通过品种改良、高接换优和经济林综合管理，保证特色产业优质丰产。重点扶持一批规模大、效益好、带动力强的龙头企业，认证一批龙头企业和品牌产品，深度开发国内外市场，实现特色产业提质增效。通过五年的努力，把陇南打造成全国优质核桃主产区、全国最大的花椒主产区、全国油橄榄主产区和全省中药材主产区，实现核桃、花椒产业全国最大，油橄榄产业全国最强的目标。同时，抢抓我市整体进入国列秦巴山片区集中连片特殊困难地区扶贫攻坚和“两州两市”扶贫重点的机遇，积极推进开发式扶贫，以农业产业发展带动扶贫开发，认真落实农村低保和开发式扶贫“两项制度”，积极实施整村、片带推进，大力发展劳务经济，健全和完善领导包抓、部门帮扶、社会参与的大扶贫工作格局，力争实现“两不愁、三保障”目标。二是推进工业跨越式发展。加快工业项目建设，以把陇南建成全省重要的有色冶金工业基地、水电能源基地和特色农产品加工基地为目标，发展壮大有色冶金、农特产品加工、水电能源、医药化工、建筑建材等主导产业，推动工业经济总量持续增加。重点推进甘肃厂坝有色金属有限责任公司20万吨锌冶炼、阳山金矿、礼县亚特矿业、临江20万千伏安硅铁基地、生物质能源等重大项目建设和油橄榄、茶叶、核桃等特色农产品精深加工，扩大工业经济生产能力，使锌冶炼能力达到40万吨，黄金产量达到5吨，硅铁生产能力达到30万吨以上，水泥生产能力达到600万吨，其中黄金、铅锌产量达到省内第一，使陇南成为西北重要的有色金属生产基地。切实加强工业园区建设，按照产业集聚、项目集中、集约发展的总体要求，制定优惠政策，产业向园区集中，企业向园区入驻，扶持资金向园区倾斜，使工业集中区成为区域经济新的增长极。科学编制园区发展规划，进一步优化主体功能区划布局，整合各方面生产建设要素，大力建设水、电、路等基础设施，积极开展园区专项招商，大力推进技术创新，引进低碳环保产业企业，把西成经济开发区建成甘肃南部有色冶金工业、建材工业、农特产品加工业基地和关中-天水经济开发区的延伸区，把徽县工业集中区建成承接产业转移、资源循环利用的示范园区，并在其它县区建立承载县域经济发展的新平台。着力整合工业资源要素，积极引进战略合作伙伴，进一步加大铅锌、黄金、硅铁、建材、农特产品加工等产业的资源整合、资产重组和嫁接兼并力度，培育一批管理水平高、核心竞争力强的现代化大企业、大集团，力争培育3户市场竞争力强、有知名品牌、主营业务收入达15亿元以上的上市公司，3户市场竞争力较强、主营业务收入达10亿元以上的企业集团，5户主营业务收入达5亿元以上的优势企业，10户主营业务收入达亿元的骨干企业，30户主营业务收入达5000万元以上的重点企业，支撑全市经济社会发展。三是培育壮大第三产业。全力打造旅游五大精品景区，完善提升四大名牌景区，争创两个国家5A级景区，六个国家4A级景区；积极打造精品旅游线路，配套完善旅游服务设施，切实提升旅游产业发展水平，真正使旅游成为支撑经济发展的支柱产业和人民群众更加满意的现代服务业。积极引进大型连锁商贸企业和现代管理技术，支持保险、法律、会计、金融租赁、信用担保、咨询、广告等中介咨询服务业和文化创意、商务会展、服务外包等现代服务业发展，加快发展生产性、生活性等基础服务业，改造提升传统服务业，切实提高服务业的整体水平。利用兰渝铁路、武都至罐子沟和成县至武都高速公路等重大交通项目建成运营的机遇，加快建设市级物流中心和各县县城物流中心、乡镇配送中心，推进农产品批发市场建设和改造，实施“万村千乡”等市场工程，发展农资连锁经营，不断健全完善市场流通体系；加强市场监督管理，规范市场经营秩序。加强房地产市场指导和监管，严格落实商品房价格控制措施，确保房地产业健康发展。加快培育出口创汇产业，促进有条件的特色产品扩大出口规模，实现外贸出口新突破。

（二）着力解决瓶颈制约，实现交通建设大突破。坚持把交通建设放在经济社会发展的突出位置，彻底扭转全市交通落后的被动局面，使交通建设在跨越式发展中发挥重要的支撑和保障作用。一是加快重大交通项目建设。完善重大项目建设工作协调机制，强化与国家、省有关部门的联系沟通，着力破解影响项目建设的突出问题，确保项目建设顺利推进。尽快建成武都至罐子沟高速公路、成县至武都高速公路、兰渝铁路和兰成铁路，开工并建成陇南成州民用机场，开工建设十堰至天水高速公路陇南段、渭源至武都高速公路，积极做好武都至九寨沟高速公路、天水至平凉铁路南延伸线、阳平关至九寨沟铁路前期工作，争取尽早开工建设。二是改造提升国省干线和县际公路。实施国省道和重要县际联网公路升等改造工程，提升干线公路等级，国省道达到二级公路标准，县道达到三级公路标准；建设宕昌南河至迭部腊子口、礼县至武都、礼县至武山洛门、西和至武都4条通县二级公路，实现县县通二级公路；新建康县、徽县、礼县、两当、文县高速公路连接线，使市内各县区、主要风景区与高速公路、铁路之间实现二级公路连接。三是加强农村公路建设。实现全市195个乡镇全部通沥青（水泥）路，建设通村公路通畅工程1386项8000公里，使2500个行政村通沥青（水泥）路，全面提高16000公里农村公路的管护水平。到2016年，全市将形成以武都至罐子沟、成县至武都、十堰至天水陇南段、渭源至武都高速公路和兰渝、兰成铁路为主骨架，国省干线及县际公路为主干道，农村公路为补充、运输站场为支点的遍布城乡、连接纵横、畅通快捷的综合交通运输体系，提升交通运输对经济社会发展的支撑保障能力。

（三）着力实施城镇化战略，实现城镇建设大发展。坚持加大力度，强化措施，提质扩容，提升品位，促进市区、县城、小城镇和农村集镇协调发展。一是构建以市区建设为中心，以八县县城为骨架，以建制镇为基础，以新农村建设为辐射的开放式城乡建设体系，利用有山有水气候好、森林覆盖率高的自然条件，通过总体规划修编、控制性、修建性详细规划编制和风貌改造，彰显陇南地域文化特色，创建宜居宜游的陇南山水园林新城镇。二是市区建设上在五年内分阶段建成长江大道东西延伸工程，把城市框架拉大到40平方公里，建成市级行政中心、“四馆四中心”和体育场、体育馆、南山公园等项目，加快建设完善东江新区、钟楼滩新区、吉石坝新区，完善城市路网、管网和供水、供电、供暖工程，配套建设公园、广场、学校、医院等服务设施，新建一批四星级、五星级宾馆，把市区建成功能完善、特色鲜明的政治文化中心和交通枢纽。三是在八县县城建设中把旧城改造和新区开发结合起来，创新方法解决征地拆迁难、项目落地难等突出问题，拉大城市建设框架，使建成区面积在2011年的基础上扩大一倍以上；大力实施市政设施和公共设施项目，集中建设一批山体绿化、河道治理、公园、广场项目，不断完善城市功能。四是加快哈达铺、阳坝、江洛、盐官、碧口、黄渚、杨店、长道等21个示范小城镇建设，按照各自条件，配套建设市政基础设施和公共服务设施，打造一批特色鲜明、文化韵味突出、有一定产业优势的重点旅游、工业和商贸型小城镇。五是进一步加快新农村建设步伐，高标准完成新农村规划编制工作，每年力争新建新农村190个以上，到2016年全市新农村总数达到1800个以上，基本形成城乡协调发展格局。同时，切实加强城镇建成区的规划管理，巩固建设成果，提升城镇品位。

（四）着力夯实基础设施，实现发展条件大改善。坚持重点突破、项目带动，强力推进基础设施建设，加快改善发展条件。一是加强以水利为重点的农村基础设施建设。贯彻落实2011年中央一号文件和中央水利工作会议精神，加快水利改革与发展，实行最严格的水资源管理制度，建立水资源管理三条红线，规范水能资源开发，实现人水和谐。大力开展农田水利建设，全面完成现有病险水库加固整治，加强中小河流治理，抓好各县城区、重要集镇、村社堤防工程建设及徽成盆地和“三江一水”沿岸河谷川坝区老灌区维修配套工程，开展白龙江、白水江、西汉水半山台地水浇地建设，新增和恢复灌溉面积126万亩，改善灌溉面积9万亩。大力实施农村饮水安全工程，力争到2016年全面解决农村人畜饮水安全问题。努力创造条件，积极争取水利部推行的各项改革示范试点。积极实施土地整治项目，加大梯田建设力度，加强中低产田改造，改善农业基本条件，每年建设10万亩标准农田。二是加强生态环境保护和建设。积极争取实施白龙江、白水江、西汉水及中小河流综合治理工程，继续实施生态修复、退耕还林、天然林保护、公益林补偿、长防林二期、自然保护区等工程，加强森林管护，提高植被覆盖率，着力构建长江中上游生态安全屏障。扎实推进污染减排，进一步加大重点区域污染防治力度，严格环境执法监督，促进环境质量改善。三是加强能源通信建设。加强城乡电网建设和改造，争取建设750千伏输电线路，新建徽县330千伏输变电工程，每县至少建成一座110千伏变电站，逐步形成多电源互供式供电网络。不断完善能源结构，优化水电开发，大力推广沼气、太阳能等新型清洁能源。积极推进电视网、通信网、互联网三网融合，推进政府部门信息共享和电子政务建设，为经济社会发展提供信息支持。四是加强防灾减灾工作。建立健全重大自然灾害监测、预警、预报和应急救援机制，完善抢险救灾应急救援预案，实施一批具有基础性、全局性、关键性的地质灾害防治、山洪泥石流灾害治理、城镇村庄安全防护、应急避险场所等工程，普及防灾减灾知识，全面提升应对各种灾害的能力。

（五）着力保障和改善民生，实现社会事业大跨越。坚持以人为本，全面统筹社会事业发展，大力改善民生，不断提升人民群众的幸福感和满意度。一是优先发展教育。完善义务教育投入保障机制，深化教育改革，促进学前教育、义务教育、高中阶段教育、高等教育和继续教育协调发展，积极支持陇南师专升本。整合教育资源，进一步建设完善农村寄宿制学校，调整学校布局，优化城乡教育资源配置。加强学校管理，强化校长、教师、教研队伍建设，加大督查考评力度，提升中小学教育质量，努力办人民满意的教育。二是突出发展卫生事业。深化医药卫生体制改革，健全城镇职工、城镇居民和农村居民三项医疗保障，完善市县乡村（社区）四级卫生服务网络，强化医疗卫生专业人才队伍建设，支持中医中药事业发展，加快疾病预防控制体系建设，加强妇幼卫生保健工作，全面实行基本药物制度，实现人人享有基本医疗卫生服务目标。三是大力发展文化体育事业。认真贯彻落实党的十七届六中全会精神，充分开发利用文化资源，加强公共文化体系建设，提高公共文化服务能力。引导文化团体向市场经营型转变，繁荣文艺创作，推进文化基础设施、文化基本阵地、文化产业、文化队伍“四项建设”，建设文化长廊，开展精神文明创建活动，促进文化大发展、大繁荣，努力建设文化大市。广泛开展全民健身运动，提高竞技体育水平，促进体育事业健康发展。四是着力保障和改善民生。加快建设“城乡一体、全民覆盖”的社会保障体系，不断完善城乡低保、农村五保户供养、特困户救助、灾民救助等制度，进一步扩大基本养老、基本医疗和失业、工伤、生育保险覆盖面，加大失地农民养老保险力度，努力提高社会保障能力。加快保障性住房建设和农村危旧房改造，严格控制商品房价格过快上涨。全面落实促进就业再就业的各项政策，鼓励自主创业，以创业促进就业。大力发展劳动密集型产业和服务业，多渠道、多方式增加就业岗位，积极引导大中专毕业生、复退军人就业，着力解决零就业家庭的就业问题。进一步健全劳动保障监察和劳动争议调处机制，维护劳动者特别是农民工的合法权益。五是全面做好人口工作。完善以现居住地管理为主的流动人口计划生育管理和服务体系，加强计划生育管理，稳定人口低生育水平。六是促进军民融合发展。加强全民国防教育、民兵预备役、征兵和人民防空工作，深化双拥共建，着力提升国防后备力量建设水平。

（六）着力推进机制创新，实现发展活力大增强。坚持把深化改革开放作为推动发展的内在动力，消除制约发展的体制机制障碍，切实增强发展后劲和活力。一是深入推进各项改革。继续深化行政管理体制、人事制度、医药卫生体制、财税体制等各项改革，完善政府部门服务企业发展、项目建设的长效机制，建立与高校和科研院所合作、人才培养引进、存量土地流转等机制，着力破解制约发展的瓶颈问题，为经济社会跨越式发展营造良好环境。二是积极扩大对外开放。加大对外交流和宣传推介力度，落实招商引资责任制，创新招商方式，采取节会招商、上门招商、以商招商等各种有效形式，提高招商水平，积极吸引符合国家产业政策的好项目落地陇南。加强与周边城市的交流与协作，主动融入关中—天水经济区，继续加强与深圳的后续合作，大力推进互利共赢的区域合作，形成多层次、多元化的对外开放新格局。三是大力发展民营经济。认真落实《国务院关于鼓励和引导民间投资健康发展的若干意见》，把民营经济纳入国民经济发展规划，进一步完善支持民营经济发展的配套政策，降低门槛，减轻负担，营造支持民营经济发展的良好氛围，引导民营经济在特色产业、水电能源、商贸旅游、信息服务等领域投资兴业、加快发展。大力扶持个体工商企业。健全完善民营企业信用担保体系，积极拓宽创业融资渠道，为经济社会发展注入新活力。

2012年的工作

2012年是新一届政府的开局之年。全市经济社会发展的主要预期目标是：生产总值增长13%以上，规模以上工业增加值增长18%；全社会固定资产投资增长20%以上；财政收入增长15%；社会消费品零售总额增长18%；城镇居民可支配收入增长12%；农民人均纯收入增长13%；城镇登记失业率控制在4.56%以内；人口自然增长率控制在6.8‰以内；万元生产总值综合能耗下降3.43%，全面完成年度污染减排任务。

实现上述目标，必须重点抓好以下八个方面的工作：

(一)抓好项目建设，保持投资增长。认真储备论证、争取引进和组织实施一批大项目，着力保持投资快速增长。一是抓好重大项目建设。切实解决好兰渝铁路、武都至罐子沟高速公路、成县至武都高速公路等重大项目实施中的征地拆迁问题，确保完成年度投资计划。开工建设十堰至天水高速公路陇南段、陇南成州民用机场及礼县至武都、礼县至武山洛门、宕昌南河至迭部腊子口通县二级公路。积极做好成县祁连山200万吨新型干法水泥生产线、文县临江20万千伏安硅铁生产基地、西和大桥金矿日处理1500吨矿石生产线、甘肃亚特矿业公司日处理6000吨尾渣综合回收生产线等在建项目的跟踪服务，加快建设进度，争取早日投产。二是加快地震灾后重建项目设施配套完善和“8.12”暴洪灾后重建进程。抓紧做好灾后重建项目收尾工作，加快学校、医院等公益设施的配套完善，确保及早投入使用发挥效益。扎实做好成县“8.12”暴洪灾害灾后恢复重建，加快重建项目实施进度，确保全面完成重建任务。三是加强项目前期工作。积极抓好渭源至武都和武都至九寨沟高速公路的汇报衔接，尽快完成阳山金矿、甘肃厂坝有色金属公司新建20万吨锌冶炼生产线项目前期工作，力争及早开工建设。论证储备投资千万元以上的项目50个，其中投资亿元以上的项目10个。四是加大招商引资力度。按照招商引资工作责任制的要求，采取各种方式，搞好项目洽谈和协调服务，力争全年签约资金116亿元，到位资金52亿元，合同履约率达到85%以上，项目开工率达到50%以上。

(二)加强“三农”工作，促进农民增收。全面落实各项惠农政策，切实加大投入力度，努力增加农民收入。一是加快特色农业产业开发。新发展特色农业基地面积26万亩，出栏商品牛羊35万头（只），肉蛋奶产量12万吨。加大新品种、新技术引进推广力度，完成经济林优质苗木繁育2500万株，经济林综合管理150万亩，嫁接换优150万株。新培育市级以上龙头企业10户，新建农民专业合作经济组织30个，完成订单农业40万亩。继续抓好农产品注册认证工作，年内新增无公害农产品产地认定、绿色食品认证、有机食品认证5项，申报国家地理标志产品保护3项。进一步加大特色农业示范点建设力度，努力实现特色农业产业提质增效。二是落实科技增粮措施。推广全膜双垄沟播技术18万亩、脱毒种薯125万亩，测土配方施肥360万亩，努力提高粮食单产，确保粮食总产稳定在100万吨以上。三是深入推进扶贫开发。积极争取秦巴山特殊困难地区扶贫攻坚规划项目落地实施，认真实施“五大扶贫”工程，全年实施整流域整乡推进试点10个、扶贫整村推进100个。加大农村劳动力输转力度，增加培训投入，建立公共实训基地和示范基地，提高输转劳动力素质和组织化输转水平，全年输转劳务70万人，创劳务收入66亿元，力争全市攻坚区农民人均纯收入增幅高于全省平均水平2个百分点，减少贫困人口8万人。四是加大惠农政策落实力度。按照公开、公平、公正的原则，不折不扣地落实粮食直补、良种补贴、农机具购置补贴等各项惠农政策，严格执行“一册明、一折通”和民主评议公示制度，确保各项惠农资金及时足额发放到群众手中。五是深化农村综合改革。继续推进农村土地经营权流转制度、集体林权配套改革和基层农业推广体系改革，认真落实草原生态补奖政策，全面完成畜牧兽医、农产品质量安全管理、农业行政综合执法等体制改革任务。

(三)加快工业发展，促进转型升级。以促进工业转型升级和提高竞争力为目标，扎实推进工业经济发展。一是加快工业园区建设。启动徽县工业集中区道路、水电、厂房等基础设施建设，积极开展园区招商，力争园区建设和项目引进取得实质性进展。抓紧完成西成经济开发区核心区规划审批工作，进一步抓好基础设施建设，切实提高项目承载能力和吸引力，力争在引进企业、壮大产业上有新突破。进一步完善武都吉石坝现代物流园区规划，及早开展基础设施建设，为园区发展打好基础。二是支持重点企业扩大生产。加强工业企业经济运行监测，发布运行分析报告，提供资源、市场、技术信息，引导企业按市场需求科学有序组织生产，积极开拓市场，增强产品竞争力。加大煤、电、油、运协调保障力度，支持重点企业提高生产能力。三是大力扶持中小微企业发展。认真落实扶持政策，切实减轻企业负担，积极帮助解决融资、土地等事关企业生产和发展的具体困难。加强评估、咨询、法律、会计、信息服务，促进中小微企业发展。四是切实加强节能减排。严格落实节能减排目标责任制，加强对重点行业、企业的能耗监测和污染治理，实施好环境保护项目，全面完成节能减排目标任务。严格执行国家产业政策，加快淘汰小水泥、小硅铁等高耗能、高污染企业，积极论证实施资源综合利用项目，推进循环经济发展。

(四)加强商贸旅游，促进第三产业发展。进一步加强旅游、商贸基础设施建设，不断拉动城乡消费，提升第三产业质量和效益。一是抓好旅游服务设施建设。修编完善《全市旅游发展总体规划》、《县区旅游发展总体规划》和市级旅游专项规划、各大景区建设详细规划；加大五大精品景区、四大名牌景区观光步道、景观设施、停车场等公共服务设施的建设力度，争取将文县天池、西和晚霞湖、宕昌哈达铺建成国家4A级景区，年内创建星级宾馆5家；加快乡村旅游发展，建成10个旅游示范村；加强旅游服务技能培训，组织开展行业服务竞赛和餐饮大赛，着力提高服务质量和水平。全市接待游客人数达到450万人，旅游综合收入达到21亿元。二是抓好城乡市场体系建设。认真实施“万村千乡市场工程”、“新网工程”，加快花椒、核桃、油橄榄、中药材、茶叶专业市场建设，改造提升农贸市场，新增农村集贸市场17个、农家店400个、直营店60个、配送中心6个。三是积极扩大消费。认真落实扩大消费优惠政策，全面实施“家电下乡”、“家电以旧换新”，积极开展农超对接，确保城乡消费稳定增长。支持长城果汁等农产品出口企业扩大出口规模，力争实现外贸进出口总额1380万美元以上。

(五)依托项目投资，强化基础设施。继续加强城乡基础设施建设，为经济社会发展提供强力支撑。一是加快城镇建设。尽快完善市区建设规划，合理划分市区两级城市规划建设管理职责，健全完善市级融资平台。继续推进市区东江新区、钟楼滩新区、吉石坝新区建设，完成土地统征、火车站拆迁安置和站前广场拆迁建设，建成市政广场、栈道湾白龙江大桥、北峪河西堤路等项目，开工建设五星级宾馆和西南、文化、乡企、社保四个小区等旧城改造及供水扩容改造项目，完成集中供热、莲湖广场开发续建项目，抓好吉石坝新区规划完善和路网等基础设施建设项目实施。把南山作为扩大城市空间的重要区域，完成规划编制，争取尽快开工建设基础设施和服务设施项目，依山就势建设主题公园，造福市区群众。加快长江大道东西延伸工程建设进度，力争年内建成主体工程，带动市区发展。加快各县县城道路、供排水、供热、污水处理、垃圾处理等139个市政基础设施和公共服务设施项目建设，依托项目推进以21个示范镇为重点的小城镇建设，增强综合服务带动功能。二是加快交通建设。完成242公里通乡油路、100个行政村470公里通村水泥路建设任务，开工建设200个行政村1000公里通村水泥路，使行政村公路硬化率达到30%；实施县乡公路危旧桥梁改造15座，完成国省干线沿线部分乡村公路硬化建设任务。三是加快水利建设。重点抓好白龙江干流堤防、中小河流治理、西汉水沿岸18万亩苹果基地节水灌溉和白龙江流域半山干旱区16万亩油橄榄基地上水工程等项目前期工作，编制完成《陇南市八县一区坡耕地综合治理实施方案》，加大衔接争取力度，力争年内启动实施。继续抓好水利基础设施建设，力争新增有效灌溉面积5万亩、集雨补灌4万亩、节水灌溉4万亩，新修梯田15万亩。四是加强生态环境建设。继续实施小流域综合治理、退耕还林、生态修复、天然林保护等工程，完成退耕还林、荒山造林10万亩，实施天然林保护工程管护面积1014万亩、重点公益林312万亩，小流域综合治理300平方公里。编制完成陇南市重金属污染治理、农村环境连片整治、文县天池生态保护项目实施方案，争取年内启动实施。建立健全山洪泥石流灾害预警预报机制，健全信息共享平台，提高防灾减灾水平。五是加快电力通信建设。实施农网改造升级工程，完成徽县榆树、两当城关变电站改造和武都安化至佛崖、西和鱼磨至草关、城关至大桥线路改造等35千伏及以下农网改造升级工程。加强通讯网络建设，着力提高全市农村、边远地区信息网络覆盖率。

(六)抓好财税金融，提高保障能力。加强财税征收监管和金融调控，充分发挥财政、金融对经济社会发展的保障和调节作用。一是努力促进财政增收。加强财源建设，大力培育新的税收增长点，不断增强市县财政自我发展能力。坚持依法治税，强化对重点行业、重点企业的税收征管和非税征缴，做到应收尽收，确保全面完成财政收入任务。加大各类财政转移支付资金争取力度，申报争取更多项目资金。二是保障重点支出。调整优化财政支出结构，严格压缩和控制一般性支出，重点保证“三农”、社会事业、民生实事、主导产业和重点项目的投入，提高公共财政保障能力。三是深化财政管理体制改革。深化部门预算改革和非税收入管理改革，进一步扩大国库集中支付改革市级试点范围，规范行政事业单位国有资产管理，努力提高财政科学化、精细化、规范化管理水平。加强对重大财政政策执行、民生项目实施、强农惠农资金落实、扩大内需资金使用情况的监督检查，加大专项资金绩效评价力度，切实提高财政资金使用效益。四是改善金融服务。进一步加强银政、银企合作，组织开展银企合作对接活动，引导金融机构加大对优势产业、中小企业、重点项目的信贷支持力度，积极支持和服务地方经济发展。逐步引进全国或区域性商业银行在陇南设立分支机构，鼓励发展小额贷款公司和新型农村金融机构，切实解决“贷款难”的问题。

(七) 发展社会事业，保障和改善民生。坚持以人为本，加强民生事业和公共服务项目建设，促进各项社会事业全面进步。一是努力提高教育质量。深入推进教育体制机制改革，深化课程改革，完善考核评价体系，强化学校管理，加强教师队伍建设，进一步提高中小学教育质量，扩大学前教育资源，促进职业教育内涵发展。二是大力推进科技进步。支持市级科研院所发展，不断增强创新能力。积极引导和鼓励企业申报中小企业创新基金、科技重大专项等科技引导项目。加强科技人员管理，调动科技人员积极性，大力推广先进适用技术，抓好农民科技普及培训，扎实推进科技特派员创业行动。三是大力发展文化、广播电视、体育事业。加大对文化事业的投入，加强文化队伍建设，力争实现文化信息资源共享工程村级服务点和农家书屋全覆盖，推动文化馆、图书馆、博物馆等公共文化设施向社会免费开放。开展文艺奖评选活动，促进文艺繁荣。继续推进广播电视“村村通”建设工程。广泛开展全民健身活动，办好市第七届运动会。四是加强卫生和人口与计划生育工作。继续深化医药卫生体制五项改革，实现新农合“一卡通”全覆盖；加强公共卫生工作，大力发展中医药事业。扎实推进生殖健康服务进家庭行动和全国利益导向政策体系建设示范区创建工作，切实稳定低生育水平。五是千方百计扩大就业。积极引导自主创业和灵活就业，进一步做好大中专毕业生、城镇下岗失业人员、城镇新增劳动力、复转退伍军人等重点人群的就业培训和就业促进工作。加大小额担保贷款扶持就业创业力度，促进创业带动就业。六是加快建立全民覆盖的社会保障体系。九县区全面启动城乡居民社会养老保险试点，推进基本医疗保险制度城乡一体化改革，完善城镇职工、居民基本养老和基本医疗、失业、工伤、生育保险制度，努力做到全体居民应保尽保，参保人员待遇应享尽享。进一步完善社会救助体系，鼓励社会捐助，重点抓好低收入群体和弱势群体的保障和救助工作。积极维护职工权益，在企业推行工资集体协商制度，促进职工工资随着企业发展稳步提高。七是加强“菜篮子”建设。加大投入力度，制定优惠政策，抓好新一轮“菜篮子”工程建设，建立蔬菜、畜产品运输绿色通道，满足广大群众的基本生活需求。八是做好民族宗教工作。认真实施少数民族乡村经济社会发展规划，抓好成县“各民族共同团结奋斗、共同繁荣发展示范县”建设；积极开展宗教领域普法教育和“和谐寺观教堂”创建活动。同时，重视加强全民国防教育、民兵预备役、征兵、人民防空、双拥模范县（城）创建工作；继续做好外事侨务、统计、气象、地震、测绘、档案、地方志等各项工作。

坚持改善民生办实事，2012年重点办好10件民生实事：⑴市财政筹措3000万元，对城市低保、农村低保、农村五保、重点优抚对象等困难群众给予一次性物价补贴。⑵市财政筹措2000万元，武都区、成县、宕昌县配套资金，建成花椒、核桃、中药材、茶叶4个大型现代化专业市场。⑶市财政筹措3000万元，相关县区配套资金，建成5个景区的游客接待中心，完善4个景区的旅游服务基础设施。⑷市财政筹措1500万元，各县区配套资金，新建生态文明示范村160个，完成新农村规划设计和图集1000个（包括村落基本建设风格式样及单体户型设计等）。⑸市、县财政筹措资金，解决市、县新建图书馆图书、设备配套和农村文化管理员报酬。⑹建设保障性住房3000套（其中经济适用房400套，廉租住房600套，城市棚户区改造2000套）。⑺解决10万农村人口和农村学校师生安全饮水；保障乡村3000多部应急指挥调度电话的联络畅通。⑻建设100个行政村的道路通达工程。⑼完成城乡劳动力职业技能培训并输转3万人次；扩大城乡居民养老保险试点范围，年内争取在现有试点县基础上全面推行；新增城镇就业1万人。⑽建成泊车100辆以上的城区停车场11个；建成国省干线公路沿线便民公厕及垃圾收集点100处。

(八)加强社会管理，促进社会和谐稳定。深入研究社会管理规律，完善管理机制，推进社会管理创新，促进社会和谐稳定。一是加强应急管理。完善重大事项风险评估和突发事件应急管理机制，建立健全响应迅速、处理高效的公共突发事件应急体系，完善应急处置预案，积极开展应急演练，提高突发事件监测预警、风险控制、应急救援和保障的水平。二是强化安全生产。建立安全生产长效机制，进一步加大安全生产投入，突出抓好煤矿、非煤矿山及尾矿库、危险化学品、道路交通、消防、建筑施工、重点项目建设等重点领域的专项整治,坚决防止重特大事故发生, 组建矿山应急抢险救援队伍，确保人民群众生命财产安全。抓好食品药品质量监管，让老百姓吃上放心食品，用上放心药品。加强对流动人口、重点人群、复杂场所和虚拟社会的管理。三是加强物价调控。建立陇南市价格调节基金制度，加强粮食、食用油、肉禽蛋、蔬菜等农产品储备供应,强化价格监督检查，力争全市居民消费价格总水平涨幅控制在5%左右。四是扎实开展社会治安综合治理。狠抓平安陇南建设，完善社会治安防控体系，依法打击各类违法犯罪活动，提高人民群众的安全感。深入开展“六五”普法教育，强化法律服务和法律援助，提高全社会的法制意识。五是有效做好信访工作。创新信访工作机制，坚持领导干部接待群众制度，提升信访工作科学化水平。创新群众工作方式方法，完善矛盾纠纷排查预警、调解处置机制，建立健全人民调解、行政调解、司法调解工作机制，协调处理好各方面利益关系。

各位代表，实现未来五年的宏伟目标，完成2012年的各项任务，对政府工作提出了新的更高的要求，我们必须坚持不懈地加强政府自身建设，进一步改革创新，扎实工作，努力做到为人民服务、对人民负责、受人民监督、让人民满意。一是增强跨越发展的信心，担负起新的历史使命，通过加强学习，强化理论武装，解放思想，转变观念，开阔眼界，胸怀大局，善于用世界眼光判断大势，用战略思维谋划发展，用统筹理念推动工作，着力增强工作的主动性、预见性和创造性，探索加快发展的新途径，努力建设创新型政府！二是自觉接受人大及其常委会的法律监督、工作监督和政协的民主监督，健全行政决策机制，完善重大事项集体决策、专家咨询、社会公示与听证以及行政复议、信访、申诉制度，严格依照法定权限和程序行使权利、履行职责，依法管理经济社会事务，切实规范行政行为，努力建设法治型政府！三是切实树立真抓实干作风，紧紧围绕任期目标和当年任务，联系实际作决策，咬定目标不放松，转变作风抓落实，加快节奏求实效，确保政令畅通、执行有力；坚持把更多的时间和精力向基层倾斜，做到调查研究在一线、问题解决在一线，用心想事、用心谋事、用心干事，开短会、讲短话、发短文，说实在话、做实在人、干实在事，形成埋头苦干、少说多干的良好政风；采取措施治懒、治慵、治散，强化行政问责，开展效能监察，严肃认真处理行政不作为行为，敢抓敢管、敢于负责、勇于担当，努力建设效能型政府！四是把服务基层、服务群众、服务发展作为崇高的政治责任和精神追求，在发展的过程中妥善处理事关人民群众切身利益的具体问题，使人民群众在发展中得到更多实惠，增强履行职责的自觉性，下大力气规范工作制度、工作流程、工作行为、办公秩序、协调机制，简化办事程序，提高服务水平。严格执行首问责任制、限时办结制、责任追究制，建立行政审批项目动态清理机制，丰富政务公开内容，扩大政务公开范围，规范政务大厅运行机制，大力推广电子政务，为群众提供便捷高效服务，努力建设服务型政府！五是始终牢记“两个务必”，保持人民公仆本色，做到为民、务实、清廉；认真落实党风廉政建设责任制和领导干部廉洁从政若干规定，完善惩治和预防腐败制度体系，建立健全运行监督机制，规范土地开发、政府采购、项目资金安排、国有资产转让、重大工程建设招投标管理；进一步健全行政监察、审计监督等内部监督机制，切实纠正部门和行业不正之风；坚持勤俭节约，反对奢侈浪费，坚决抵制拜金主义、享乐主义，查处惩治各种腐败行为，努力建设廉洁型政府！

各位代表，风正海阔，自当扬帆破浪、一往无前；任重道远，更需策马挥鞭、奋勇争先。全力推进陇南科学发展、跨越发展，是时代赋予我们的重任，也是全市人民寄予我们的重托。让我们在市委的坚强领导下，把人民的期望化作前进的动力，把崇高的使命化作积极的行动，群策群力，团结拼搏，开拓创新，奋发有为，为推进陇南经济社会跨越式发展而努力奋斗！
